# Correlates of preparedness for caregiving of poststroke patients: a meta-analysis

**DOI:** 10.3389/fneur.2025.1465962

**Published:** 2025-05-29

**Authors:** Chen Li, Yanhui Fang, Huan Wang, Bingqin Lin, Yuyao Xie, Yuanyuan He, Dawei Dong, Dongxiang Zheng

**Affiliations:** ^1^Department of Neurology and Stroke Center, The First Affiliated Hospital of Jinan University, Jinan University, Guangzhou, China; ^2^School of Nursing, Jinan University, Guangzhou, China; ^3^Department of Neurology, The Affiliated Shunde Hospital of Jinan University, Foshan, China

**Keywords:** stroke, caregiver, caregiver preparedness, correlate, meta-analysis

## Abstract

**Background:**

Given that stroke is a sudden, traumatic medical crisis and a chronic condition, identifying factors associated with caregiver preparedness is particularly important for poststroke caregivers. Therefore, we carry out this study to identify correlates of preparedness for caregiving for poststroke patients. To examine correlates of preparedness for caregiving in poststroke patients.

**Methods:**

A systematic review and meta-analysis was performed according to the PRISMA 2020 guidelines. We searched six English databases and three Chinese databases for studies published from the establishment of the database to May 2023. The quality of the evidence was assessed using the Agency for Healthcare Research and Quality scale. Statistical software R studio was used for statistical analysis.

**Results:**

Thirteen studies were included in the meta-analysis. Caregivers for poststroke patients reported relatively low-to-moderate level of caregiver preparedness. Our meta results showed that demographics characteristics of both poststroke patients and caregiver, stroke-related variables and psychological variables were associated with caregiver preparedness. And subgroup analysis stroke type contributes to heterogeneity in caregiver gender, age and relationship, caregiver type contributes to heterogeneity in caregiver experience.

**Conclusion:**

The level of caregiver preparedness ranges from low to moderate and is influenced by multiple factors. The findings may inform tailored strategies for enhancing preparedness in stroke caregivers.

**Systematic review registration:**

https://www.crd.york.ac.uk/PROSPERO, identifier CRD42021249641.

## 1 Introduction

According to the most recent Global Burden of Disease (GBD) estimates, stroke was the second leading cause of death and third leading cause of disability globally in 2019. There were approximately 12.2 million cases of stroke, 143 million disability-adjusted life-years (DALYs) lose due to stroke, and 6.6 million stroke-related deaths worldwide. If the current trend continues, the costs of stroke care will likely increase steeply over the next 20 years unless practical measures to prevent stroke are effectively created implemented (1). Most stroke patients have functional and self-care impairments due to hemiplegia or other disabilities. As the number of stroke survivors increases, the need for caregiving will increase as well.

Stroke is a sudden and traumatic medical crisis for patients and their families, and informal caregivers are often forced to assume caregiving responsibilities within days of the event (2, 3). Due to the chronic nature of stroke, the potential for health deterioration, and the subtle manner in which complications develop, most stroke survivors rely on unprepared informal caregivers for support after discharge from hospital (4). In general, informal caregivers often perform caregiving without any training or education.

Caregiver preparedness is defined as a caregiver’s perception of their level of readiness to manage patient emergencies, attend to the physical and emotional needs of patients, and provide patient health care (5). Insufficient caregiver preparedness often results in psychological and physical health issues for the caregiver and poststroke patients. For caregivers, insufficient caregiver preparedness is associated with caregiver burden, depression and compromised quality of life (6, 7). In terms of poststroke patients, insufficient caregiver preparedness is associated with lower quality of life, worse recovery and higher risk of hospital readmission (8–10).

Several studies have designed interventions to improve caregiver preparedness, and positive intervention effects on preparedness have been observed in stroke and cancer (11, 12). These positive outcomes may be related to the fact that the intervention was designed to target modifiable factors affecting the level of caregiver preparedness.

As preparedness is associated with better outcomes in patients and the caregiver population and can be modified via interventions, it is crucial to understand the factors that contribute to poststroke caregiver preparedness. Identifying factors relevant to stroke caregiver preparedness may help motivate clinicians to develop and implement interventions for post-stroke patients and caregivers. There is a gap in the evidence regarding correlates of caregiver. Therefore, this study conducted a systematic review and meta-analysis to identify risk factors for caregiver preparedness by assessing demographic factors, stroke-related factors, and psychosocial factors. Our study was designed to systematically review the literature on the correlates of caregiver preparedness in poststroke patients.

## 2 Methods

### 2.1 Reporting and protocol registration

This study was conducted following the guidelines of the Cochrane Collaboration and Preferred Reporting Project for Systematic Review and Meta-Analysis (PRISMA) Guidelines (Supplementary File 1), (13) and was retrieved on PROSPERO^1^ (number: REDACTED).

### 2.2 Search strategy

The search was carried out in six English databases [PubMed, Cochrane Library, Embase, Web of Science, PsycINFO and Cumulative Index to Nursing and Allied Health Literature (CINAHL)] and three Chinese databases [the China National Knowledge Infrastructure (CNKI), Weipu (VIP) and Wanfang Data from inception to March 2023]. In addition, the search also included the search engine and relevant research references. Medical Subject Heading (MeSH) terms and free terms were combined in the search strategy, which is presented in Supplementary File 2.

### 2.3 Inclusion and exclusion criteria

The inclusion criteria were developed based on the PICOS principle: P: informal caregiver of patients diagnosed with stroke; I: caregiver preparedness, measured using valid and reliable self-report scales; O: independent quantitative measures regarding caregiver preparedness and at least one other variable (such as demographic variables, disease-related variables and psychosocial variables); S: cross-sectional or longitudinal design. Studies published in peer-reviewed journals or scholarly journals were included. Only English and Chinese studies were included. When multiple articles were based on the same dataset, the one with more complete outcomes, higher research quality or the largest sample size was included for further analysis.

The exclusion criteria were as follows: (i) studies did not report relevant outcomes; (ii) editorials, reviews, case reports, letters, and comments; and (iii) the data could not be extracted or assessed by contacting the corresponding author.

Two researchers screened the titles and abstracts of the retrieved studies to determine if they met the inclusion/exclusion criteria. Then, the full texts of the potentially eligible studies were retrieved. Any disagreements were resolved by consulting a senior researcher.

### 2.4 Data extraction

The following data were extracted from the included studies and entered into a standardized electronic data entry sheet: first author, publication year, country, study setting, sample size, stroke type, caregiver type, study design, eligibility requirements, caregiver preparedness identification and level, and main correlates for associated factors. Baseline data for longitudinal study were extracted.

Three researchers were involved in the data extraction process. Two researchers extracted key outcome statistics and analyzed them independently, while a third researcher validated the initial data and findings. Reviewing the original study and check the data to solve disagreements.

### 2.5 Quality appraisal

Two researchers independently performed the methodological quality of each included studies by using the Agency for Healthcare Research and Quality (AHRQ) scale (14). Quality ratings are classified as follows: 0–3 points (low-quality), 4–7 points (medium-quality), and 8–11 points (high-quality). Discrepancies in risk assessments were resolved through consensus-based discussions, with a third reviewer being consulted to arbitrate unresolved disagreements.

### 2.6 Statistical analysis

The meta-package in the statistical program R studio was used to perform all quantitative statistical analyses. First, to investigate caregiver preparedness and the correlation between related factors, the Pearson correlation coefficient (*r*), the Spearman correlation coefficient, (15) the standardized regression coefficient (*β*) and odds ratio and the value of a multivariate analysis (standardized *β*) were calculated (16, 17). Second, we used Pearson correlation coefficients transformed through Fisher *z*-transform to calculate the pooled *z*-values (18).

*I*^2^ statistics were used to measure heterogeneity. *I*^2^ values of <25%, ~50%, ~75%, and ~100% were considered to indicate different levels of heterogeneity (19). Research studies used a fixed effects model to estimate the *z* value if *I*^2^ was less than 50%; otherwise, a random effects model was used. To identify potential sources of heterogeneity, subgroup analyses were performed.

## 3 Results

**Figure 1:**
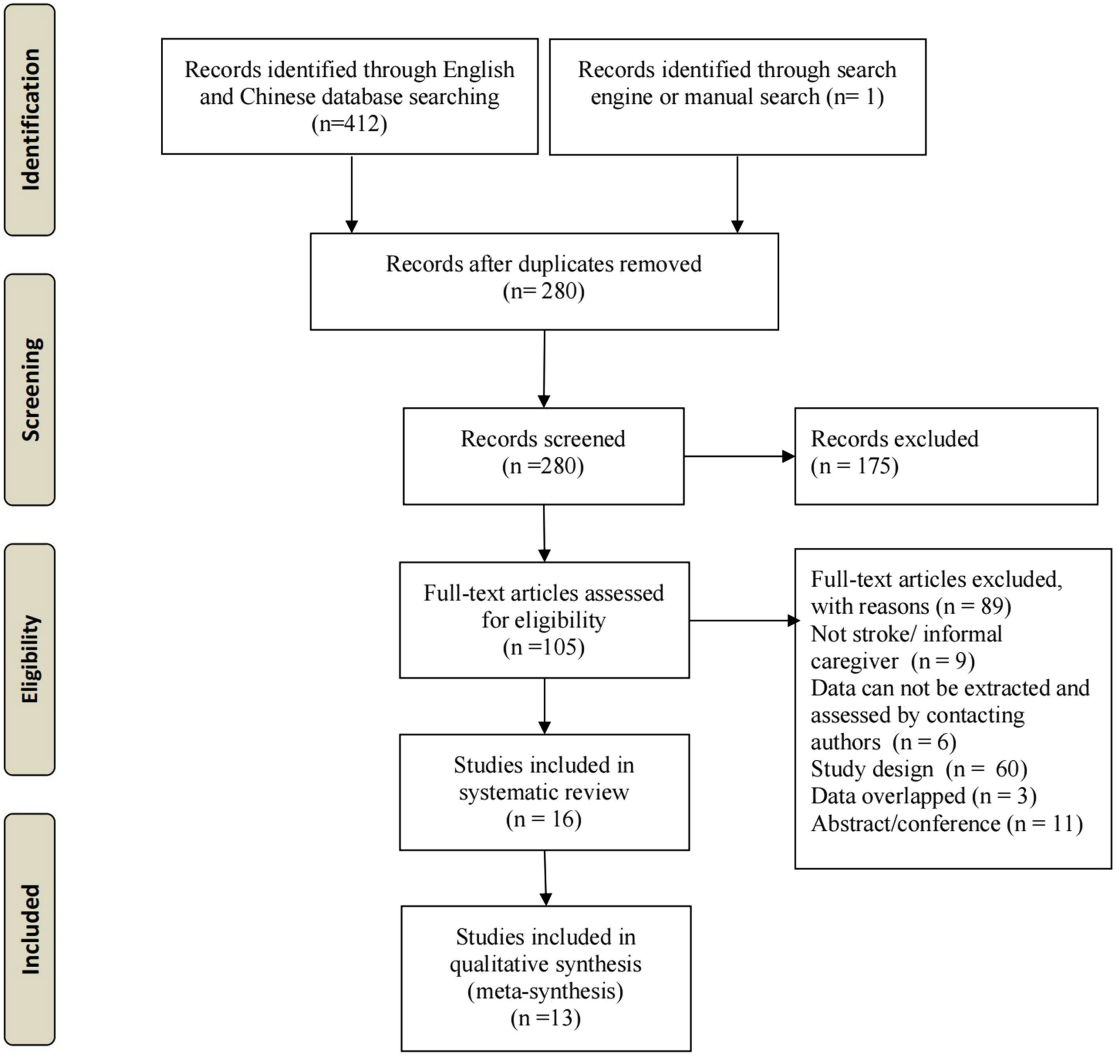
Flow diagram of studies included and excluded at each stage of review.

Our initial search yielded 413 studies. After deleting duplicates and excluding irrelevant studies based on titles and abstracts, 106 full-text articles were evaluated based on the inclusion criteria. Ultimately, 13 studies were included in the meta-analysis (Figure 1).

### 3.1 Characteristics of included studies

Regarding the geographic location of the study, 11 studies were carried out in China (20–30), one study each was carried out in America (31), and Nigeria (32). All studies were carried out in the past 5 years. The sample size of caregivers ranged from 102 to 306. The details of the included articles are shown in Table 1.

**Table 1:** Characteristics of included studies.

Author/year	Country	Study setting	Sample size	Stroke type	Caregiver	Study design	Caregiver sex (male/female)	Caregiver age (M ± SD)	Caregiver preparedness identification	Preparedness level (M ± SD)	Ahrq Score
Camicia et al. 2021 (31)	USA	NP	183	Stroke	Family caregivers	Cross-sectional	111/72	66.90 ± 13.00	CPS	NP	8
Onu et al. (2022) (32)	Nigeria	Hospital	200	Stroke	Family caregivers	Cross-sectional	60/140	40.91 ± 8.90	CPS	NP	8
Geng (2020) (20)	China	Hospital	231	Initial stroke	Family caregiver	Cross-sectional	88/143	63.56 ± 14.38	CHCR	52.58 ± 11.689	7
He et al. (2019) (21)	China	Hospital	102	Young and Middle-aged stroke	Spouse	Cross-sectional	41/61	49.16 ± 11.86	CPS	12.87 ± 5.39	7
Hou (2021) (22)	China	Hospital	105	Middle-aged stroke	Spouse	Cross-sectional	34/71	36.60 ± 10.23	CPS	13.01 ± 5.32	7
Liu et al. (2018) (24)	China	Hospital	200	Initial stroke	Informal caregiver	Cross-sectional	75/125	NP	CPS	13.99 ± 4.95	7
Liu et al. (2020) (23)	China	Hospital	306	Initial Stroke	Family caregivers	Cross-sectional	112/194	45.69 ± 11.8	CPS	14.42 ± 5.12	8
Lv and Dong (2018) (25)	China	Hospital	110	Initial stroke	Family caregiver	Cross-sectional	44/66	47.51 ± 14.37	CPS	20.43 ± 2.3	5
Song et al. (2020) (26)	China	Hospital	205	Stroke	Informal caregiver	Cross-sectional	82/123	NP	CPS	17.42 ± 5.76	6
Sun (2020) (27)	China	Hospital	292	Stroke	Informal caregiver	Cross-sectional	101/191	NP	CPS	18.75 ± 6.04	7
Tian et al. 2020 (28)	China	Hospital	223	Stroke	Family caregiver	Cross-sectional	71/152	48.36 ± 12.21	CPS	15.31 ± 4.87	7
Wang (2021) (29)	China	Hospital	220	Initial stroke	Family caregiver	Cross-sectional	74/146	49.45 ± 11.06	CPS	18.28 ± 6.08	7
Yang et al. (2022) (30)	China	Hospital	300	AIS	Family caregivers	Cross-sectional	133/167	NP	CPS	16.23 ± 2.35	6

AIS, acute ischemic stroke; CPS, Caregiver Preparedness Scale; CHCR, Caregiver Home Care Readiness; AHRQ, the Agency for Healthcare Research and Quality.

The mean quality assessment scores for cross-sectional studies were 6.9 (AHRQ). The cumulative score of the quality assessment of included studies for the meta-analysis were presented in Table 1, and the details in Supplementary File 3.

### 3.2 Sample characteristics

All studies included stroke patients. Five studies stated that they only include initial stroke patients, and two studies included young and middle-aged stroke patients. For caregivers, the 13 included studies had a total of 1,026 male caregivers and 1,651 female caregivers. Specifically, eight examined were family caregivers, third examined were informal caregiver and two examined were spouse caregivers. Informal caregivers were middle aged and had a low-to-moderate level of caregiver preparedness.

The measures of caregiver preparedness included the Caregiver Preparedness Scale (CPS) and Caregiver Home Care Readiness (CHCR) scale. The CPS was developed by Archbold et al. (5) and is the most widely used instrument to assess caregiver preparedness. The scale consists of 8 items answered on a 5-point Likert-type scale. The CHCR scale was used by one study and consists of 19 items answered on a 5-point Likert-type scale (20). The CPS and CHCR have been shown to have acceptable validity and reliability. The characteristics of the included studies are listed in Table 1.

### 3.3 Study synthesis

The meta-analysis results of 13 studies and subgroup analysis for the correlates of caregiver preparedness are presented in Tables 2, 3 and the forest plot of each correlate is presented in Supplementary File 4. Nineteen variables were used for the meta-analysis, including sociodemographic characteristics of poststroke patients (gender and marital status), sociodemographic characteristics of caregivers (gender, age, marital status, education, occupation, monthly income, health condition, cohabiting with the patient and number of caregiver), disease-related characteristics (relationship, care time per day, caregiver experience and care ability and), and psychosocial factors (depression, positive aspects, disease uncertainty and adults attachment). Five variables were used for subgroup analysis, including sociodemographic characteristics of caregivers (gender, age and monthly income), disease-related characteristics (relationship and caregiver experience).

**Table 2:** Meta-analysis of the correlations between caregiver preparedness and demographic/stroke-related/psycho-social variables.

Correlates	*N*	Heterogeneity test		Model	Pooled *z* value	95%CI
		*I*^2^ (%)	*P*			
Demographic variables						
Stroke patient						
Gender (male)	537	80	0.02	Random	−0.08	−0.27, 0.11
Marital status (single)	526	84	0.01	Random	−0.04	−0.25,0.17
Caregiver						
Gender (male)	1756	89	<0.01*	Random	−0.04	−0.18,0.09
Age (young)	1,218	97	<0.01*	Random	0.63	0.39, 0.79
Marital status (unmarried)	526	0	0.49	Fixed	0.10	0.02,0.17
Education (high)	506	0	0.87	Fixed	0.89	0.87,0.91
Occupation (unemployed)	506	0	0.34	Fixed	0.53	0.47,0.59
Monthly income (low)	1,257	99	<0.01*	Random	−0.64	−0.91,0.02
Health condition (good)	845	98	<0.01*	Random	0.62	0.25,0.84
Cohabiting with the patient	522	0	0.63	Fixed	0.11	0.03,0.20
Number of caregiver	1,018	52	0.10	Random	0.25	0.17,0.34
Disease-related variables						
Relationship	1,549	73.7	<0.01*	Random	0.31	0.21, 0.39
Caregiver experience	1,536	93	<0.01*	Random	0.41	0.23,0.55
Care time/day	597	97	<0.01*	Random	−0.29	−0.67, 0.20
Care ability	534	87	<0.01*	Random	0.50	0.31,0.66
Psycho-social variables						
Depression	403	96	<0.01*	Random	−0.48	−0.76,−0.05
Disease uncertainty	616	66	0.05	Random	−0.55	−0.65,−0.44
Positive aspects	402	0	0.34	Fixed	0.55	0.48,0.62
Attachment avoidance	207	0	0.91	Fixed	−0.24	−0.37,−0.11
Attachment anxiety	207	0	0.97	Fixed	−0.32	−0.44,−0.19

*p < 0.0001.

**Table 3:** Subgroup meta-analysis of the correlations between caregiver preparedness and demographic/stroke-related factors.

Correlates	Subgroup	*N*	Heterogeneity test		Model	Pooled *z* value	95%CI
			*I*^2^ (%)	*P*			
Caregiver							
Gender (male)	Initial stroke	957	88.1	<0.01*	Random	0.18	−0.01; 0.35
	Stroke	799	28.7	0.24	Fix	0.09	0.00; 0.17
Age (young)	Initial stroke	726	93.1	<0.01*	Random	0.97	0.68; 1.27
	Stroke	492	0	0.83	Fix	0.39	0.30; 0.48
Monthly income (low)	Initial stroke	957	99.5	<0.01*	Random	−0.88	−1.82; 0.07
	Stroke	300	NA	NA	Random	−0.24	−0.36; −0.13
Disease-related variables							
Relationship	Initial stroke	737	76.1	0.01	Random	0.27	0.14; 0.40
	Stroke	812	0	0.98	Fix	0.40	0.32; 0.48
Caregiver experience	Initial stroke	737	96.7	<0.01*	Random	0.57	0.17; 0.97
	Stroke	799	66.3	0.03	Random	0.31	0.19; 0.44
	Family caregiver	837	87.3	<0.01	Random	0.38	0.18; 0.57
	Spouse	207	0	0.85	Fix	0.41	0.27; 0.54
	Informal caregiver	492	98.5	<0.01	Random	0.54	−0.21; 1.29

NA, not applicable; *p < 0.000.

#### 3.3.1 Sociodemographic variables (stroke)

Two sociodemographic variables (gender and marital status of stroke patients) of poststroke patients were included in the meta-analysis to examine their correlation with caregiver preparedness.

According to Table 2, the gender of stroke patients (*z* = −0.08, 95% CI, −0.27, 0.11, *p* = 0.02) marital status of stroke patients (*z* = −0.04, 95% CI, −0.25, 0.17, *p* = 0.01) were not associated with caregiver preparedness.

#### 3.3.2 Sociodemographic variables (caregiver)

Six sociodemographic variables (gender, age, marital status, education, occupation, monthly income and health condition of caregivers) of poststroke caregiver were included in the meta-analysis to determine their correlation with caregiver preparedness.

According to Table 2, age (young) (*z* = 0.63, 95% CI, 0.39, 0.79, *p* < 0.01), marital status (unmarried) (*z* = 0.10, 95% CI, 0.02, 0.17, *p* = 0.01), education (high) (*z* = 0.89, 95% CI, 0.87, 0.91, *p* = 0.87), occupation of caregiver (unemployed) (*z* = 0.53, 95% CI, 0.47, 0.59, *p* = 0.34) and good health condition (*z* = 0.62, 95% CI, 0.25, 0.84, *p* < 0.01) were associated with caregiver preparedness.

Gender (*z* = −0.04, 95% CI, −0.18, 0.09, *p* < 0.01) and monthly caregiver income (*z* = −0.64, 95% CI, −0.91, 0.02, *p* < 0.01) were not associated with caregiver preparedness.

##### 3.3.2.1 Subgroup analysis

For studies reported the correlation between caregiver gender, age, monthly income and caregiver preparedness, subgroup analysis showed that stroke type (initial stroke or stroke) contributed to heterogeneity in caregiver age and gender but not in monthly income.

For stroke type, the association between gender and caregiver preparedness was not observed in both subgroups: initial stroke (*z* = 0.18, 95% CI, −0.01, 0.35, *I*^2^ = 88.1%), stroke (*z* = 0.09, 95% CI, 0.00; 0.17, *I*^2^ = 28.7%).

For stroke type, the association between age and caregiver preparedness was significant in both subgroups: initial stroke (*z* = 0.97, 95% CI, 0.68, 1.27, *I*^2^ = 93.1%), stroke (*z* = 0.39, 95% CI, 0.30; 0.48, *I*^2^ = 0%).

For stroke type, the association between monthly income and caregiver preparedness was not observed in one subgroup: initial stroke (*z* = −0.88, 95% CI, −1.82, 0.07, *I*^2^ = 99.5%), while significant in the other subgroup: stroke (*z* = −0.24, 95% CI, −0.36, −0.13, *I*^2^ = not applicable).

#### 3.3.3 Disease-related variables

Six disease-related caregiver variables (relationship/care time per day/number of caregivers/caregiver experience/care ability and cohabiting with the patient) were included in the meta-analysis to examine their correlation with caregiver preparedness.

According to Table 2, the relationship (*z* = 0.31, 95% CI, 0.21, 0.39, *p* < 0.01), the number of caregivers (*z* = 0.250, 95% CI, 0.17, 0.34, *p* = 0.10), caregiver experience (*z* = 0.41, 95% CI, 0.23, 0.55, *p* < 0.01), care ability (*z* = 0.50, 95% CI, 0.31, 0.66, *p* < 0.01), and cohabiting with the patient (*z* = 0.11, 95% CI, 0.03, 0.20, *p* = 0.63) were associated with caregiver preparedness.

However, care per day (*z* = −0.29, 95% CI, −0.67, 0.20, *p* < 0.01) was not associated with caregiver preparedness.

##### 3.3.3.1 Subgroup analysis

For studies reported the correlation of between relationship, caregiver experience and caregiver preparedness, subgroup analysis showed that stroke type (initial stroke or stroke) contributes to heterogeneity in relationships, while caregiver type (family caregiver, spouse or informal caregiver) contributes to heterogeneity in caregiving experience.

For stroke type, the association between relationship and caregiver preparedness was significant in both subgroups: initial stroke (*z* = 0.27, 95% CI, 0.14; 0.40, *I*^2^ = 76.1%), stroke (*z* = 0.40, 95% CI, 0.32; 0.48, *I*^2^ = 0%).

For caregiver type, caregiving experience was significantly associated with caregiver preparedness among: family caregiver, or (*z* = 0.38, 95% CI, 0.18; 0.57, *I*^2^ = 87.3%), spouse (*z* = 0.41, 95% CI, 0.0.27; 0.54, *I*^2^ = 0%), care experience was associated with caregiver preparedness. However, for informal caregivers, the association between relationship and caregiver preparedness was not observed (*z* = 0.54, 95% CI [−0.21, 1.29], *I*^2^ = 98.5%).

#### 3.3.4 Psychosocial variables

Four psychosocial variables (depression/positive aspects/disease uncertainty/adult attachment among caregivers) were included in the meta-analysis to examine their correlation with caregiver preparedness.

According to Table 2, depression (*z* = −0.48, 95% CI, −0.76, −0.05, *p* < 0.01) and disease uncertainty (*z* = −0.55, 95% CI, −0.65, −0.44, *p* = 0.05) were associated with caregiver preparedness. The positive aspects (*z* = 0.55, 95% CI, 0.48, 0.62, *p* = 0.34) and dimensions of attachment (*z* = −0.24, 95% CI, −0.37, −0.11/*z* = −0.32, 95% CI, −0.44, −0.19, *p* > 0.05) were not associated with caregiver preparedness.

## 4 Discussion

In this study, we advance our understanding of the level of caregiver preparedness of poststroke caregivers and the correlates of caregiver preparedness from the perspective of demographic (poststroke patients and caregivers), disease-related characteristics and psychosocial variables.

There is an accepted conceptualization of and standard measurement tool for caregiver preparedness. The CPS is widely used to assess caregiver preparedness, which has led to consistency across our results. However, the medium and high level of heterogeneity of our results may be due to variations in grouping methods and local conditions.

Caregivers for poststroke patients reported relatively low-to-moderate level of caregiver preparedness. The level of caregiver preparedness for poststroke caregivers was consistent with patients with heart failure or disabilities (6, 10).

### 4.1 Socio-demographic variables (stroke)

The age and marital status of poststroke patients were examined in the meta-analysis, and our study showed that the age and marital status of patients were not related to caregiver preparedness. One study (33) showed that caregivers of stroke survivors with functional disabilities have severer caregiver burden, may have negative impacts on preparedness. Further studies should collect more demographic data for poststroke patients to identify more potential correlates.

### 4.2 Socio-demographic variables (caregiver)

Younger age and healthier status demonstrate significant positive associations with caregiver preparedness levels. This pattern may be mediated through enhanced physical capacity and resilience in executing caregiving tasks (34). Notably, unmarried and unemployed individuals in caregiving roles exhibit elevated preparedness metrics, potentially attributable to greater availability of temporal resources and expendable energy reserves required for sustained care provision. A greater number of assistants in caregiving and cohabiting with the patient may have a lower level of caregiver burden, which may have a positive effect on caregiver preparedness (35).

Caregivers often provide care services without adequate knowledge and information about stroke care and without any training or education (10, 36). Well-educated caregivers have higher levels of preparedness because they have more opportunities to absorb knowledge and skills and access more comprehensive information and medical help (37).

### 4.3 Disease-related variables

Caregivers in our study who had previous caregiving experience had higher caregiver preparedness. Due to unexpected diagnoses of illness or trauma in a family member caregivers often take on care without any preparation (10). Inexperienced caregivers often lack knowledge about stroke, which affects their ability to judge and manage emergencies. Past experience appears to provide a cumulative advantage in predicting future caregiving roles (38).

Multiple studies have conflicting results regarding the time burden of preparation and care. A research report suggests that PCS scores are not related to the time burden of care, such as the duration or time spent on care (10). This means that even though caregivers spend most of their time caring for patients, it does not mean they are fully prepared for the role. To guarantee higher caregiver preparedness, the amount of time spent per day on caregiving should be individualized.

### 4.4 Psychosocial variables

We also found that caregivers with depression and uncertainty were negatively associated with caregiver preparedness. This finding was consistent with previous studies (6, 10, 39, 40). Informal caregivers reported higher levels of depression when they had a high degree of uncertainty (41). One study showed that caregiver uncertainty was independently associated with caregiver depressive symptoms (42). Higher resilience was relevantly associated with lower uncertainty and depressive symptoms (43, 44). Interventions aimed at improving resilience and reducing depression and uncertainty may affect caregiver preparedness.

Positive aspects of the caregiver will encourage them to deal with their situation (45), which may contribute to a high level of caregiver preparedness. It is essential to assess caregiver preparedness for their role and the need for support to build positive aspects to maintain their role.

In contrast to our results, the attachment results did not show a significant relationship between attachment type and caregiving readiness (46). The differences between the studies may be due to demographic differences.

### 4.5 Limitations and strengths

There are some limitations to this study. First, the cross-sectional design of the included studies hindered hinders the determination of causal relationships between relevant factors and caregiver preparedness. Second, although subgroup analysis found stroke type and caregiver type contribute significant heterogeneity, limited original studies still confine the interpretation of the summarized evidence. Third, most of the included studies were carried out in China, thus limiting the generalizability of our results to other countries. Additional research should be conducted.

However, this systematic review utilized a rigorous methodological process to explore the factors associated with caregiver preparedness. Evidence summarized from this study informs subsequent research.

### 4.6 Implications

Assessing the preparedness of caregivers during hospitalization and providing timely follow-up support at home is crucial for enhancing their acquisition of new skills and promoting their adaptation to the home environment through inpatient rehabilitation.

The strategy of strengthening the preparedness of the caregivers is crucial for reducing the burden on caregiver and improving care quality. One scoping review suggested caregiver needs knowledge, training, and psychosocial support to care for patients (47). Two systematic reviews found that psychoeducation and nurse-driven interventions may be used as a useful strategy to improve caregiver preparedness (11, 48). It would be valuable for future research to explore training program or psychoeducation in improving caregiver preparedness.

Inconsistencies across studies suggest that the impact of relevant factors on caregiver preparedness is complex. Multidimensional assessment of caregiver preparedness of poststroke patients and provide sufficient support to assist caregivers in fulfilling their responsibilities.

The limited availability of sociodemographic data and the absence of disease-related information on poststroke patients hindered our ability to gather additional insights into factors associated with caregiver preparedness. Future research should prioritize investigating this area, as it may offer valuable insights for assessing caregiver preparedness in poststroke patients.

## 5 Conclusion

Caregiver preparedness at low-to-moderate levels is influenced by multiple factors, such as demographics (stroke and caregiver), stroke-related characteristics (caregiver), and psychological characteristics. To alleviate these negative outcomes and enhance the overall quality of care, early identification of caregiver preparedness and its associated factors through screening can facilitate timely support for informal caregivers in need.

## Data Availability

The original contributions presented in the study are included in the article/Supplementary material, further inquiries can be directed to the corresponding authors.

## Footnotes

^1^ https://www.crd.york.ac.uk/prospero/
