# Supplementary material for: Correlates of preparedness for caregiving of poststroke patients: a meta-analysis
Source: Front Neurol. 2025 May 29;16:1465962. doi: 10.3389/fneur.2025.1465962 (PMC12158704; doi:10.3389/fneur.2025.1465962)
Supplement: Supplementary file 1 [file Supplementary_file_1.docx]

**Supplementary File 1 Searching strategies**

| **Pubmed** | | |
| --- | --- | --- |
| #1 | (stroke[MeSH Terms]) OR (strokes) OR (Cerebrovascular Accident) OR (Cerebrovascular Accidents) OR (CVA(Cerebrovascular Accident)) OR (CVAs (Cerebrovascular Accident)) OR (Cerebrovascular Apoplexy) OR (Apoplexy, Cerebrovascular) OR (Vascular Accident, Brain) OR (Brain Vascular Accident) OR (Brain Vascular Accident) OR (Vascular Accidents, Brain) OR (Cerebrovascular Stroke) OR (Cerebrovascular Strokes) OR (Stroke, Cerebrovascular) OR (Strokes, Cerebrovascular) OR (Apoplexy) OR (Cerebral Stroke) OR (Cerebral Strokes) OR (Cerebrovascular Strokes) OR (Cerebrovascular Strokes) OR (Stroke, Cerebral) OR (Strokes, Cerebral) OR (Stroke, Acute) OR (Acute Stroke) OR (Acute Strokes) OR (Strokes, Acute) OR (Cerebrovascular Accident, Acute) OR (Acute Cerebrovascular Accident) OR (Acute Cerebrovascular Accidents) OR (Cerebrovascular Accidents, Acute) | 434,021 |
| #2 | (caregivers[MeSH Terms]) OR (Caregivers) OR (Carers) OR (Care Givers) OR (Spouse Caregivers) OR (Caregiver, Spouse) OR (Family Caregivers) OR (Caregivers, Family) OR (Informal Caregivers) OR (Caregivers, Informal) OR (relatives) | 3,086,951 |
| #3 | (Preparedness) OR (readiness) | 83,641 |
| #4 | #1 AND #2 AND #3 | 149 |
| **Web of science** | | |
| #1 | TS=(stroke OR strokes OR Cerebrovascular Accident OR Cerebrovascular Accidents OR Cerebrovascular Accident OR Cerebrovascular Accident OR Cerebrovascular Apoplexy OR Apoplexy, Cerebrovascular OR Vascular Accident, Brain OR Brain Vascular Accident OR Brain Vascular Accident OR Vascular Accidents, Brain OR Cerebrovascular Stroke OR Cerebrovascular Strokes OR Stroke, Cerebrovascular OR Strokes, Cerebrovascular OR Apoplexy OR Cerebral Stroke OR Cerebral Strokes OR Cerebrovascular Strokes OR Cerebrovascular Strokes OR Stroke, Cerebral OR Strokes, Cerebral OR Stroke, Acute OR Acute Stroke OR Acute Strokes OR Strokes, Acute OR Cerebrovascular Accident, Acute OR Acute Cerebrovascular Accident OR Acute Cerebrovascular Accidents OR Cerebrovascular Accidents, Acute) | 612,723 |
| #2 | TS=(Caregivers OR Carers OR Care Givers OR Spouse Caregivers OR Caregiver, Spouse OR Family Caregivers OR Caregivers, Family OR Informal Caregivers OR Caregivers, Informal OR relatives) | 2,450,503 |
| #3 | TS=(Preparedness OR readiness) | 88,103 |
| #4 | #1 AND #2 AND #3 | 114 |
| **Embase** | | |
| #1 | 'cerebrovascular accident'/exp OR stroke OR strokes OR (cerebrovascular AND accident) OR (cerebrovascular AND accidents) OR (cerebrovascular AND apoplexy) OR (apoplexy, AND cerebrovascular) OR (vascular AND accident, AND brain) OR (brain AND vascular AND accident) OR (vascular AND accidents, AND brain) OR cerebrovascular stroke OR (stroke, AND cerebrovascular) OR (strokes, AND cerebrovascular) OR apoplexy OR cerebral stroke OR (cerebral AND strokes) OR (cerebrovascular AND strokes) OR (stroke, AND cerebral) OR (strokes, AND cerebral) OR (stroke, AND acute) OR acute stroke OR (acute AND strokes) OR (strokes, AND acute) OR (cerebrovascular AND accident, AND acute) OR (acute AND cerebrovascular AND accident) OR (acute AND cerebrovascular AND accidents) OR (cerebrovascular AND accidents, AND acute) | 97,883 |
| #2 | 'caregivers'/exp OR caregivers OR carers OR (care AND givers) OR (spouse AND caregivers) OR (caregiver, AND spouse) OR (family AND caregivers) OR (caregivers, AND family) OR (informal AND caregivers) OR (caregivers, AND informal) | 155,781 |
| #3 | Preparedness OR readiness | 56,359 |
| #4 | #1 AND #2 AND #3 | 11 |
| **Cochrane Library** | | |
| #1 | (MeSH descriptor: [stroke] explode all trees) OR (strokes) OR (Cerebrovascular Accident) OR (Cerebrovascular Accidents) OR (CVA(Cerebrovascular Accident)) OR (CVAs (Cerebrovascular Accident)) OR (Cerebrovascular Apoplexy) OR (Apoplexy, Cerebrovascular) OR (Vascular Accident, Brain) OR (Brain Vascular Accident) OR (Brain Vascular Accident) OR (Vascular Accidents, Brain) OR (Cerebrovascular Stroke) OR (Cerebrovascular Strokes) OR (Stroke, Cerebrovascular) OR (Strokes, Cerebrovascular) OR (Apoplexy) OR (Cerebral Stroke) OR (Cerebral Strokes) OR (Cerebrovascular Strokes) OR (Cerebrovascular Strokes) OR (Stroke, Cerebral) OR (Strokes, Cerebral) OR (Stroke, Acute) OR (Acute Stroke) OR (Acute Strokes) OR (Strokes, Acute) OR (Cerebrovascular Accident, Acute) OR (Acute Cerebrovascular Accident) OR (Acute Cerebrovascular Accidents) OR (Cerebrovascular Accidents, Acute)) | 49,498 |
| #2 | (MeSH descriptor: [Caregivers] explode all trees) OR (Caregivers) OR (Carers) OR (Care Givers) OR (Spouse Caregivers) OR (Caregiver, Spouse) OR (Family Caregivers) OR (Caregivers, Family) OR (Informal Caregivers) OR (Caregivers, Informal) | 18,019 |
| #3 | (Preparedness) OR (readiness) | 5074 |
| #4 | #1 AND #2 AND #3 | 45 |
| **CINAHL** | | |
|  |  |  |
| S1 | (MH "stroke" OR TI ("strokes" OR "Cerebrovascular Accident" OR "Cerebrovascular Accidents" OR "Cerebrovascular Accident" OR "Cerebrovascular Accident" OR "Cerebrovascular Apoplexy" OR "Apoplexy, Cerebrovascular" OR "Vascular Accident, Brain" OR "Brain Vascular Accident" OR "Brain Vascular Accident" OR "Vascular Accidents, Brain" OR "Cerebrovascular Stroke" OR "Cerebrovascular Strokes" OR "Stroke, Cerebrovascular" OR "Strokes, Cerebrovascular" OR "Apoplexy" OR "Cerebral Stroke" OR "Cerebral Strokes" OR "Cerebrovascular Strokes" OR "Cerebrovascular Strokes" OR "Stroke, Cerebral" OR "Strokes, Cerebral" OR "Stroke, Acute" OR "Acute Stroke" OR "Acute Strokes" OR "Strokes, Acute" OR "Cerebrovascular Accident, Acute" OR "Acute Cerebrovascular Accident" OR "Acute Cerebrovascular Accidents" OR "Cerebrovascular Accidents, Acute")) | 43,742 |
| S2 | (MH "caregivers") OR TI ("Caregivers" OR "Carers" OR "Care Givers" OR "Spouse Caregivers" OR "Caregiver, Spouse" OR "Family Caregivers" OR "Caregivers, Family" OR "Informal Caregivers" OR "Caregivers, Informal") | 33,228 |
| S3 | (TI ("preparedness" OR "readiness")) | 5,677 |
| S4 | S1 AND S2 AND S3 | 4 |
|  | **PsycINFO** |  |
|  | (MA "stroke" ) OR TI (stroke OR strokes OR Cerebrovascular Accident OR Cerebrovascular Accidents OR Cerebrovascular Accident OR Cerebrovascular Accident OR Cerebrovascular Apoplexy OR Apoplexy, Cerebrovascular OR Vascular Accident, Brain OR Brain Vascular Accident OR Brain Vascular Accident OR Vascular Accidents, Brain OR Cerebrovascular Stroke OR Cerebrovascular Strokes OR Stroke, Cerebrovascular OR Strokes, Cerebrovascular OR Apoplexy OR Cerebral Stroke OR Cerebral Strokes OR Cerebrovascular Strokes OR Cerebrovascular Strokes OR Stroke, Cerebral OR Strokes, Cerebral OR Stroke, Acute OR Acute Stroke OR Acute Strokes OR Strokes, Acute OR Cerebrovascular Accident, Acute OR Acute Cerebrovascular Accident OR Acute Cerebrovascular Accidents OR Cerebrovascular Accidents, Acute) | 28,740 |
| #1 | (MA "caregivers" ) OR TI ("Caregivers" OR "Carers" OR "Care Givers" OR "Spouse Caregivers" OR "Caregiver, Spouse" OR "Family Caregivers" OR "Caregivers, Family" OR "Informal Caregivers" OR "Caregivers, Informal")) | 28,858 |
| #2 | (TI ("preparedness" OR "readiness") | 8474 |
| #4 | #1 OR #2 OR #3 | 4 |
| **CNKI** | | |
|  |  |  |
|  | SU%=('脑卒中'+'脑梗死'+'中风'+'脑中风'+'卒中') AND SU%=('照顾者准备度') | 60 |
| **VIP** | | |
|  | (M=脑卒中 OR 脑梗死 OR中风 OR 卒中 OR 脑中风) AND (M=照顾者准备度) | 17 |
| **Wan fang** | | |
|  | (题名或关键词:脑卒中 OR 脑梗死 OR卒中 OR中风 ) and (题名或关键词:照顾者准备度) | 8 |

**Supplementary File3 Forest plot of each correlates**


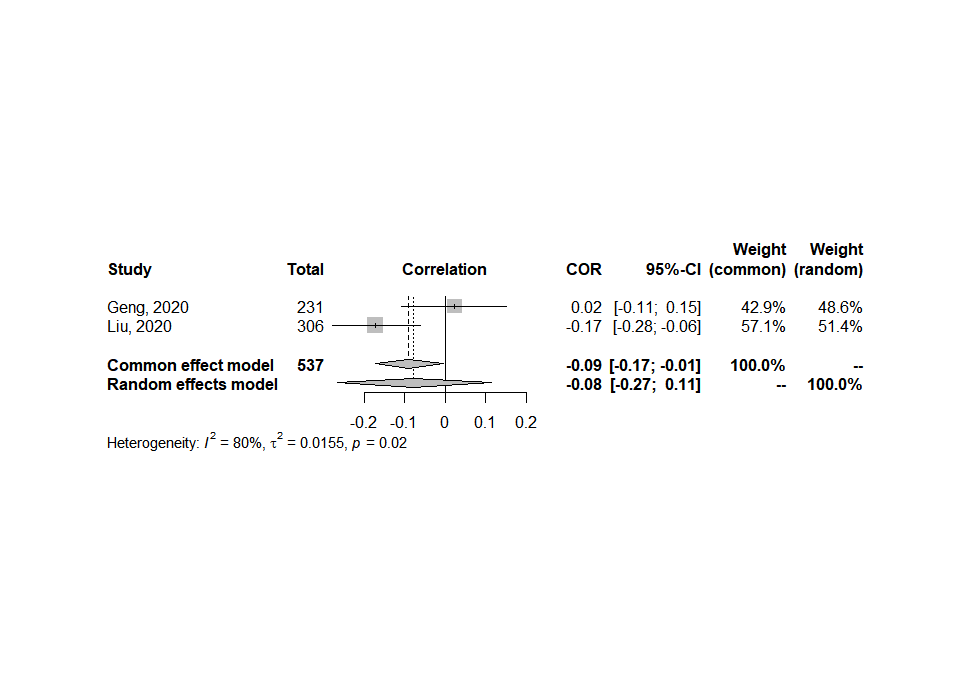


Figure 2 Forest plot: the summary z value with corresponding 95% CIs for the correlation between gender of stroke patients and caregiver preparedness.


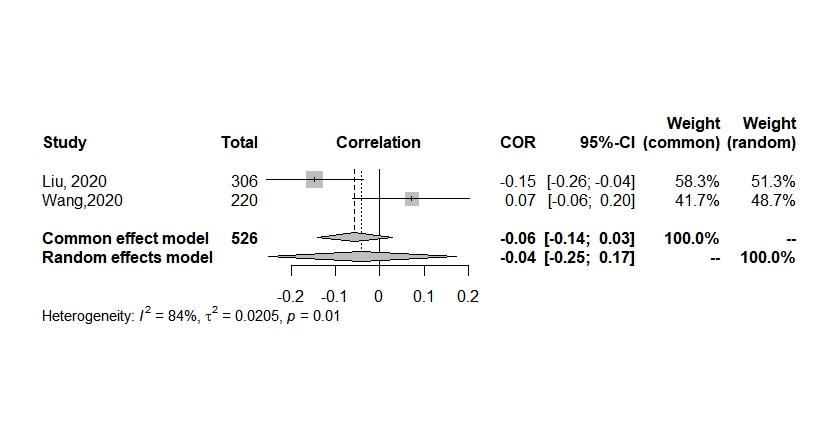


Figure 3 Forest plot: the summary z value with corresponding 95% CIs for the correlation between marriage of stroke patients and caregiver preparedness.


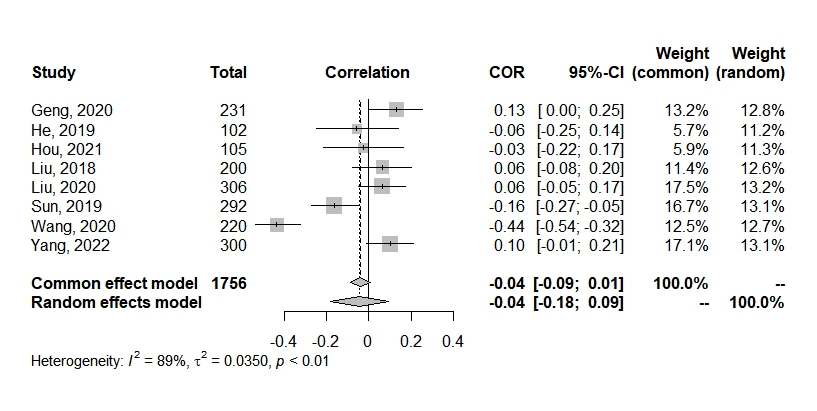


Figure 4 Forest plot: the summary z value with corresponding 95% CIs for the correlation between gender of caregiver and caregiver preparedness.


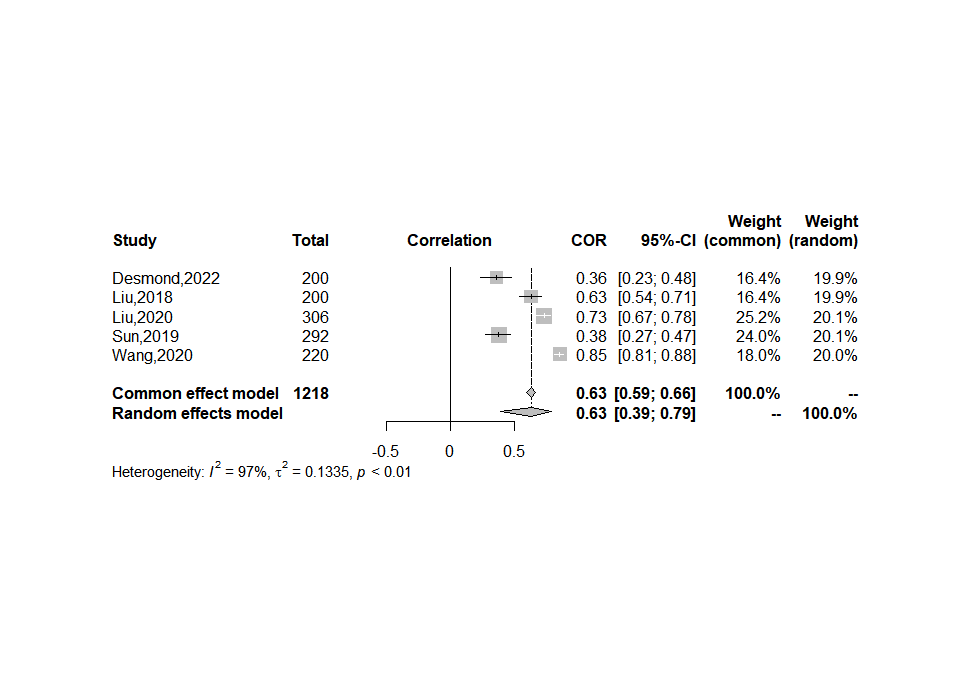


Figure 5 Forest plot: the summary z value with corresponding 95% CIs for the correlation between age of caregiver and caregiver preparedness.


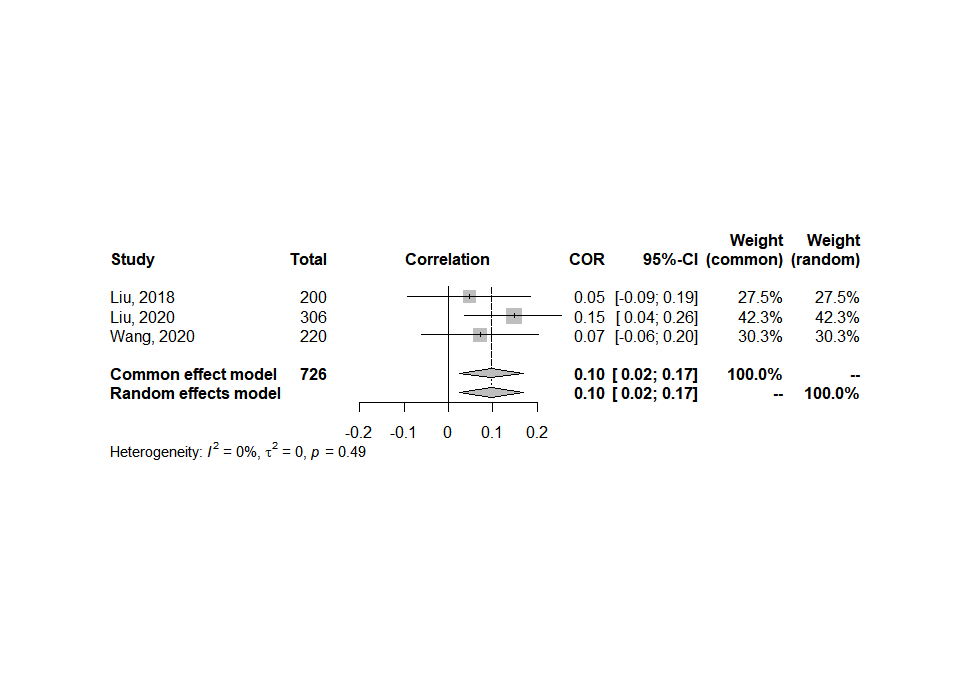


Figure 6 Forest plot: the summary z value with corresponding 95% CIs for the correlation between marriage of caregiver and caregiver preparedness.


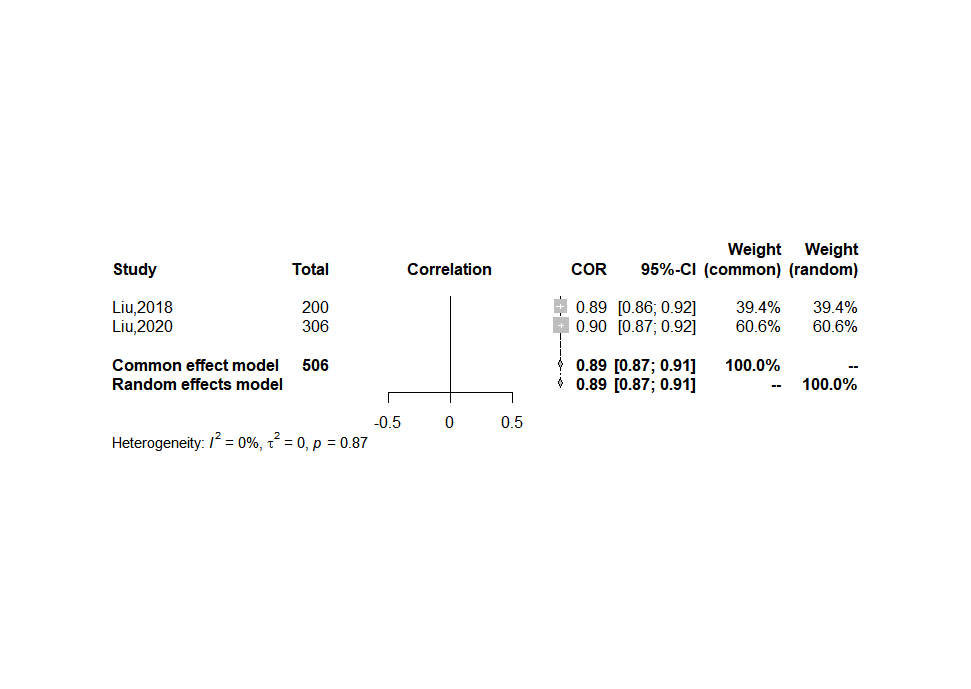


Figure 7 Forest plot: the summary z value with corresponding 95% CIs for the correlation between education of caregiver and caregiver preparedness.


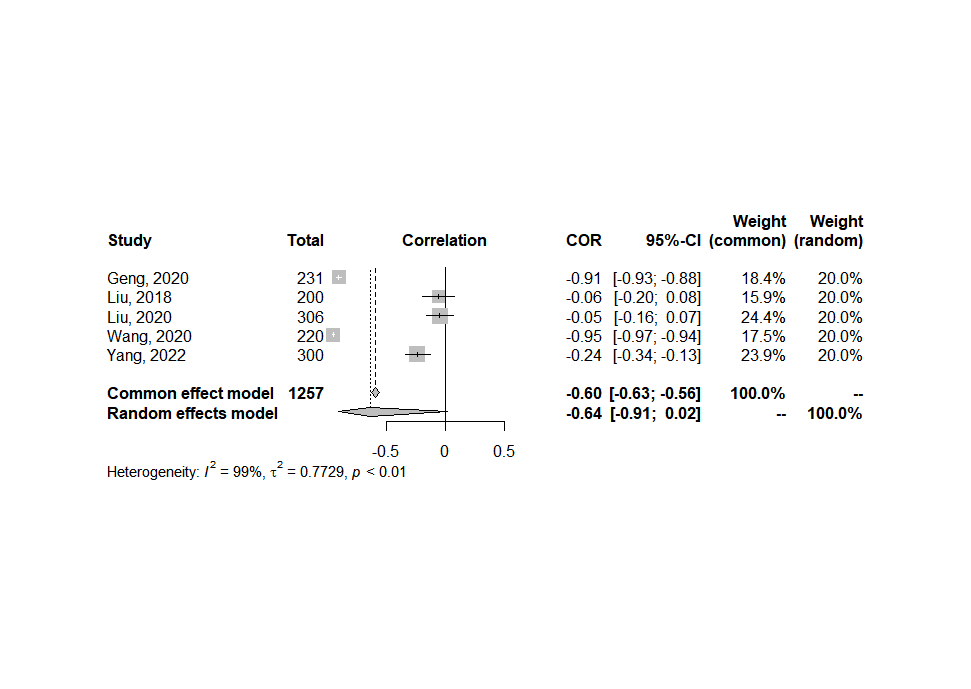
Figure 8 Forest plot: the summary z value with corresponding 95% CIs for the correlation between monthly income of caregiver and caregiver preparedness.


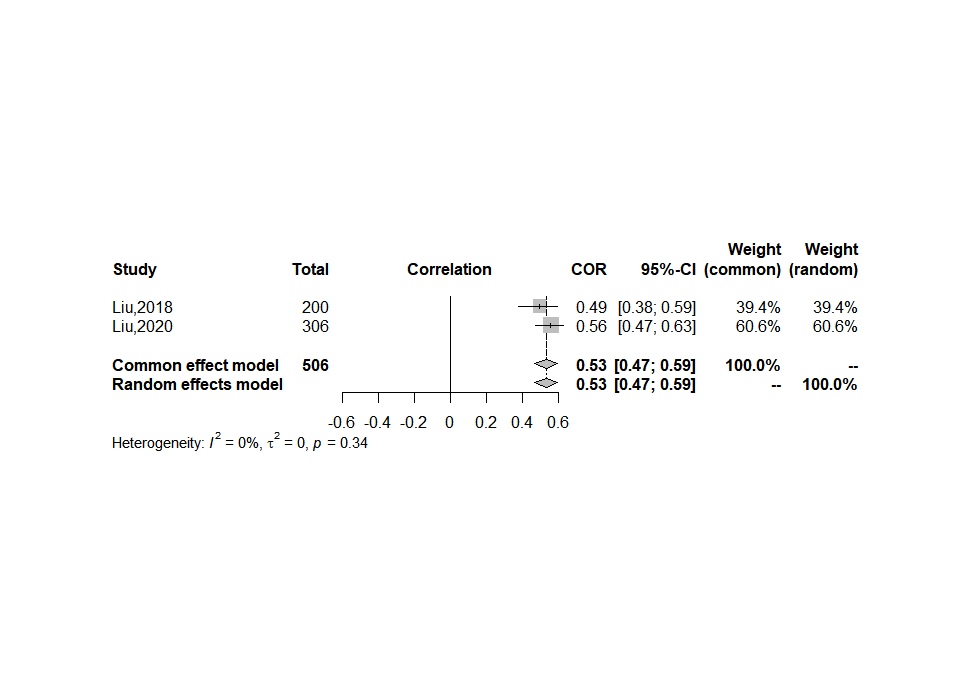


Figure 9 Forest plot: the summary z value with corresponding 95% CIs for the correlation between occupation of caregiver and caregiver preparedness.


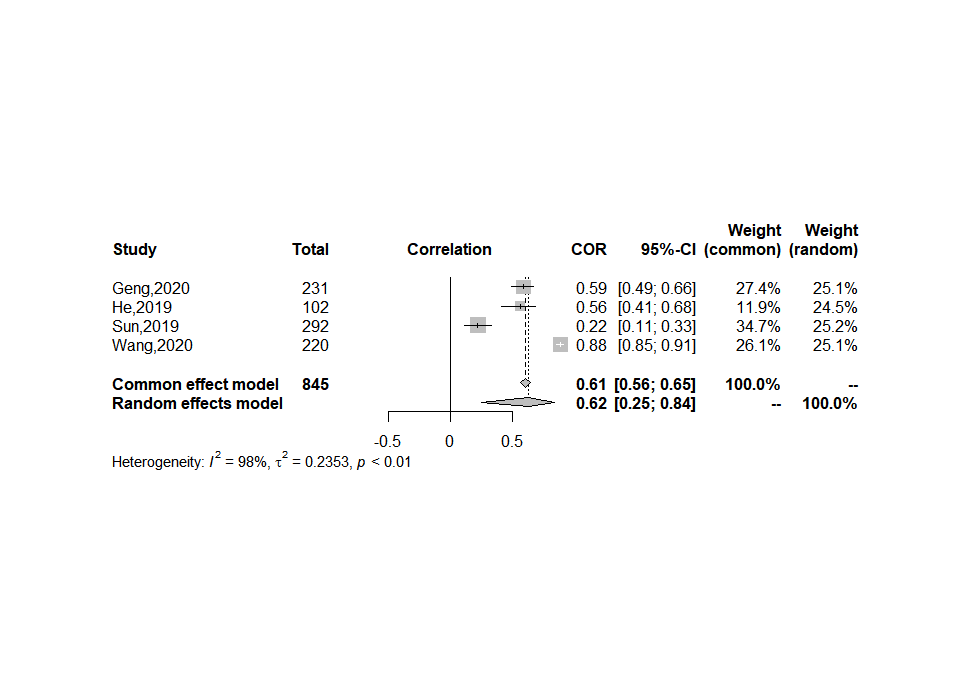


Figure 10 Forest plot: the summary z value with corresponding 95% CIs for the correlation between health condition of caregiver and caregiver preparedness.


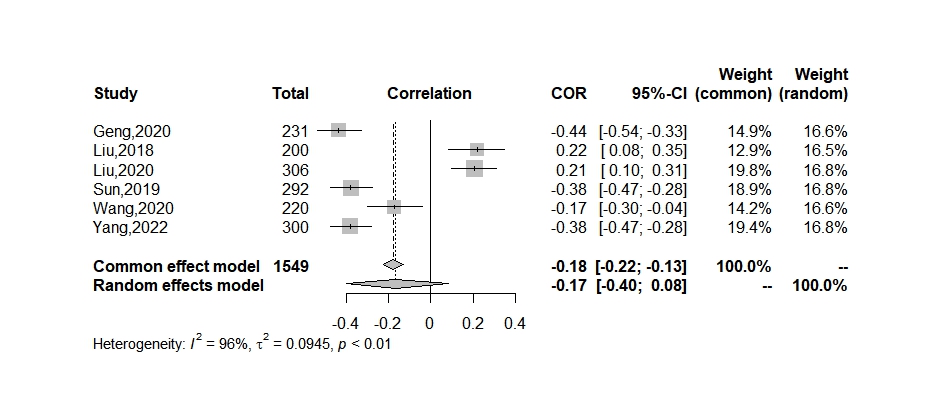


Figure 11 Forest plot: the summary z value with corresponding 95% CIs for the correlation between relationship of caregiver and caregiver preparedness.


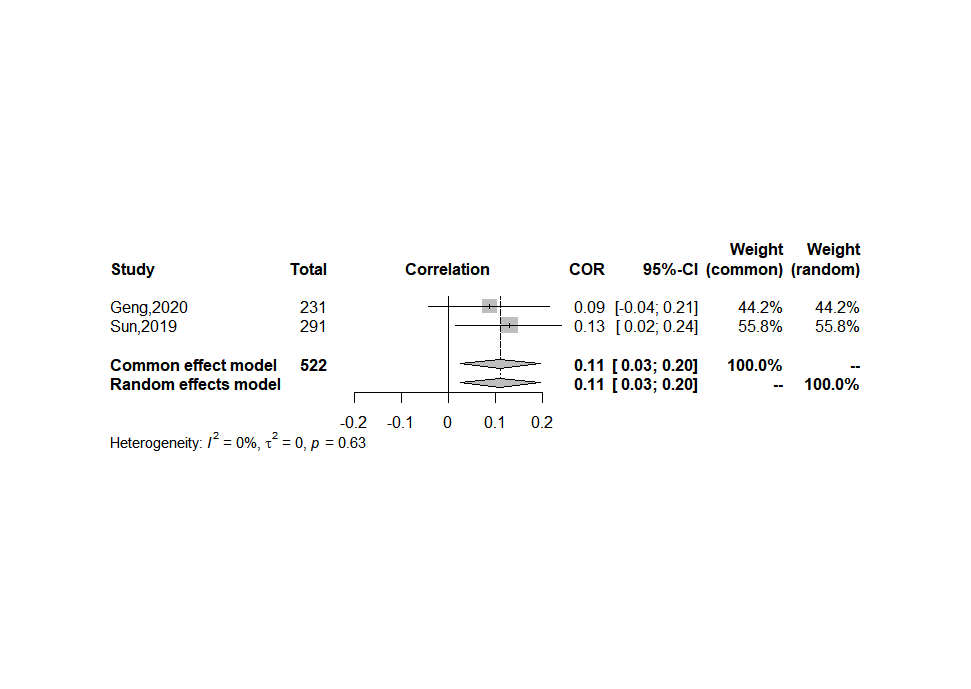
Figure 12 Forest plot: the summary z value with corresponding 95% CIs for the correlation between live with patients of caregiver and caregiver preparedness.


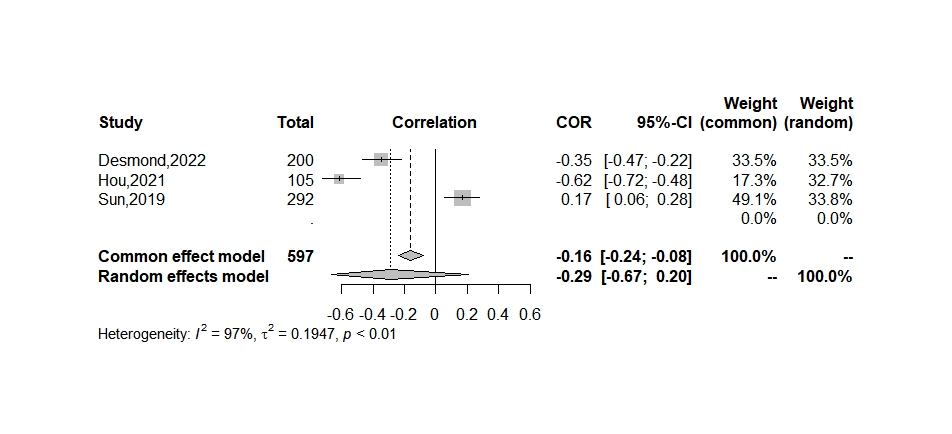


Figure 13 Forest plot: the summary z value with corresponding 95% CIs for the correlation between care time and caregiver preparedness.


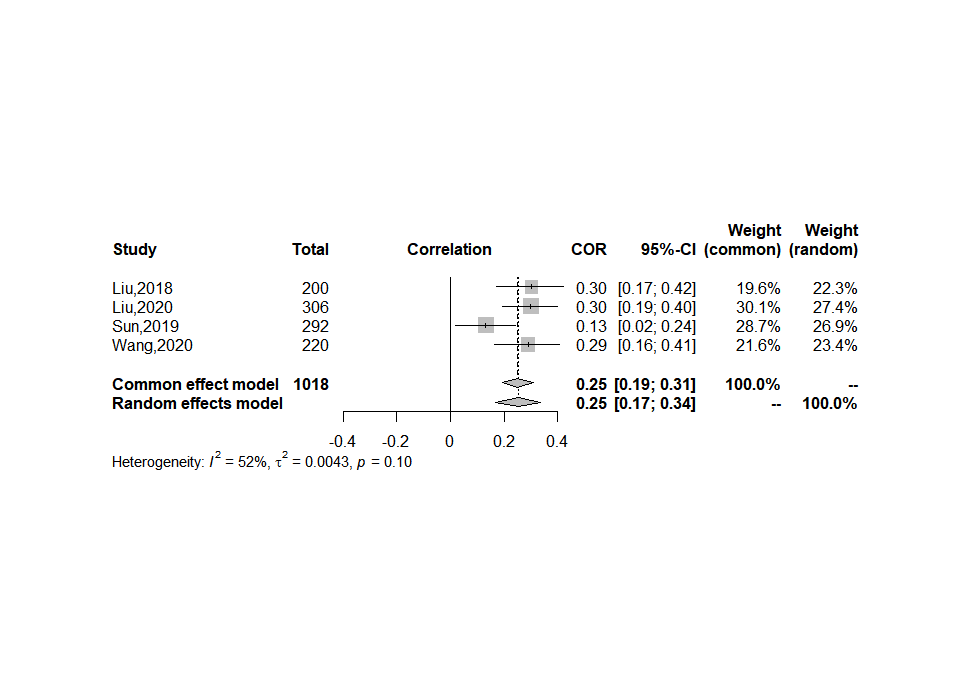
Figure 14 Forest plot: the summary z value with corresponding 95% CIs for the correlation between number of caregiver and caregiver preparedness.


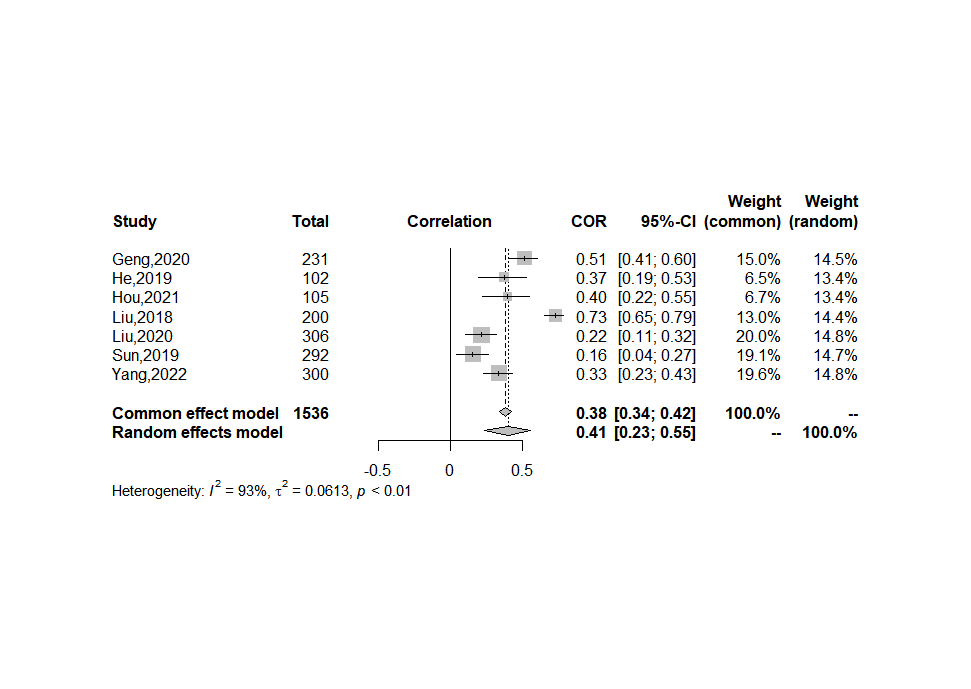


Figure 15 Forest plot: the summary z value with corresponding 95% CIs for the correlation between care experience and caregiver preparedness.


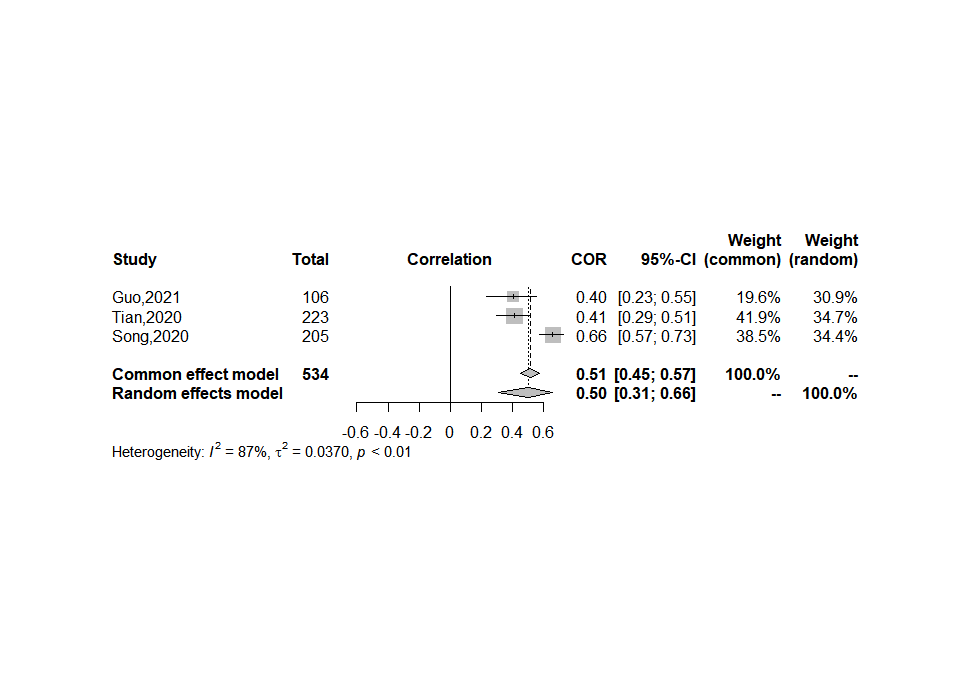


Figure 16Forest plot: the summary z value with corresponding 95% CIs for the correlation between care ability and caregiver preparedness.


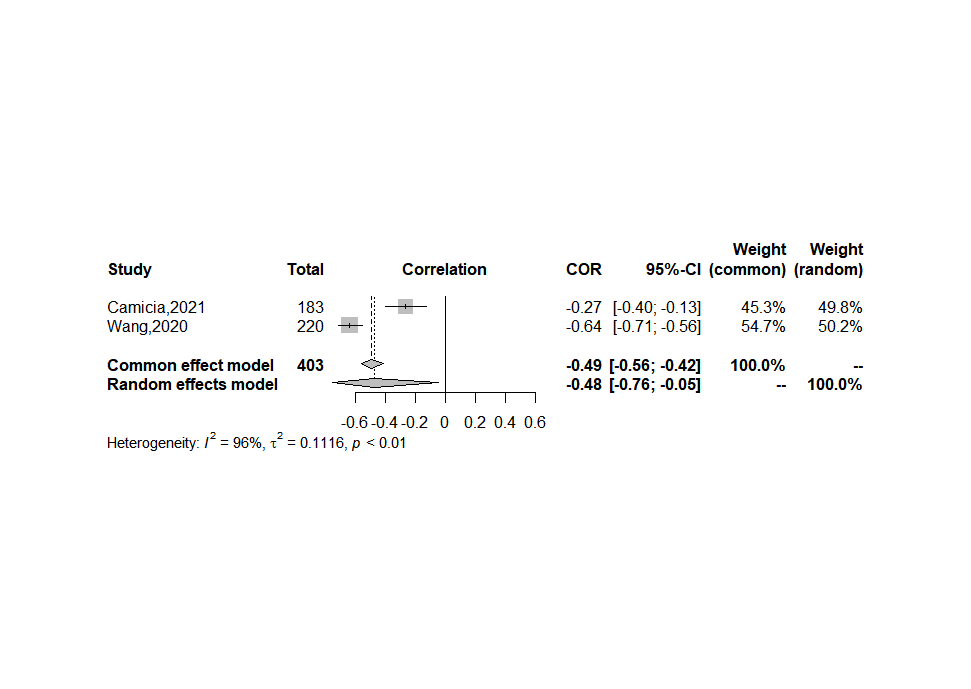


Figure 17 Forest plot: the summary z value with corresponding 95% CIs for the correlation between depression of caregiver and caregiver preparedness.


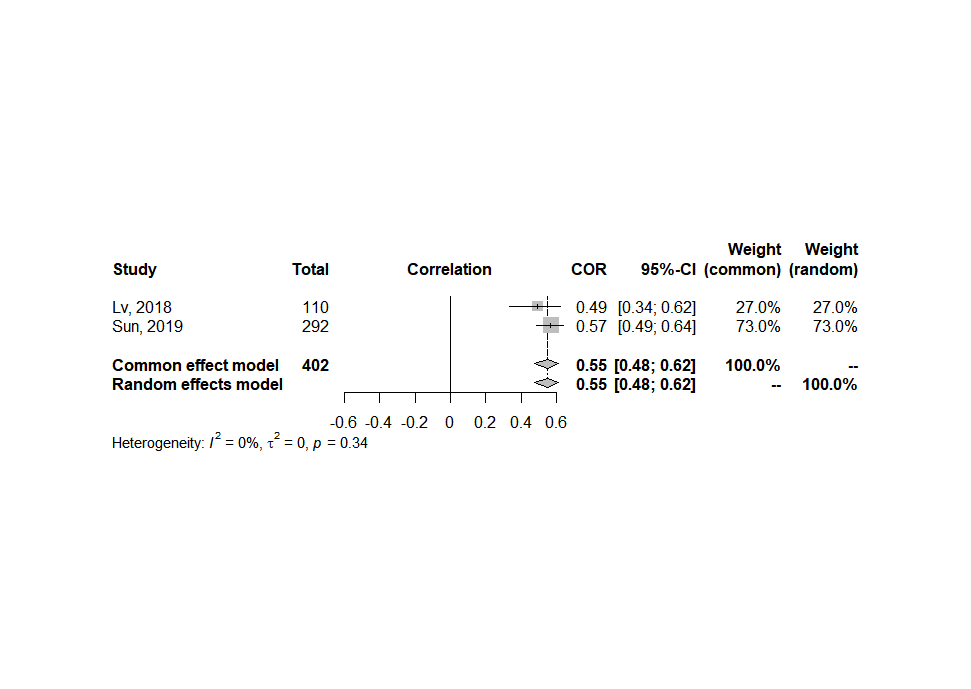


Figure 18 Forest plot: the summary z value with corresponding 95% CIs for the correlation between positive aspects of caregiver and caregiver preparedness.


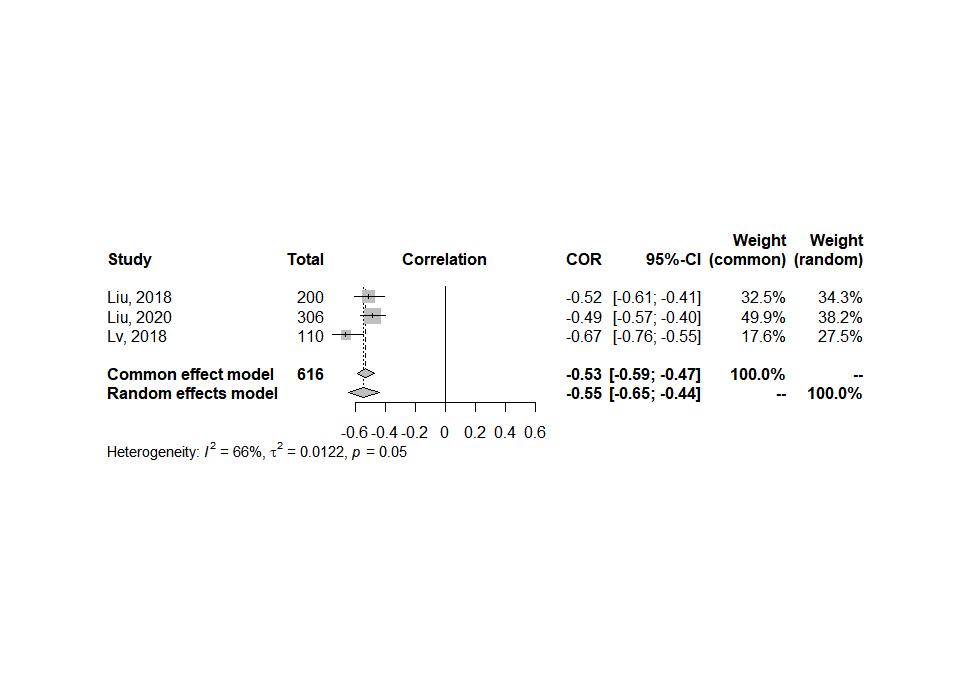


Figure 19 Forest plot: the summary z value with corresponding 95% CIs for the correlation between disease uncertainty of caregiver and caregiver preparedness.


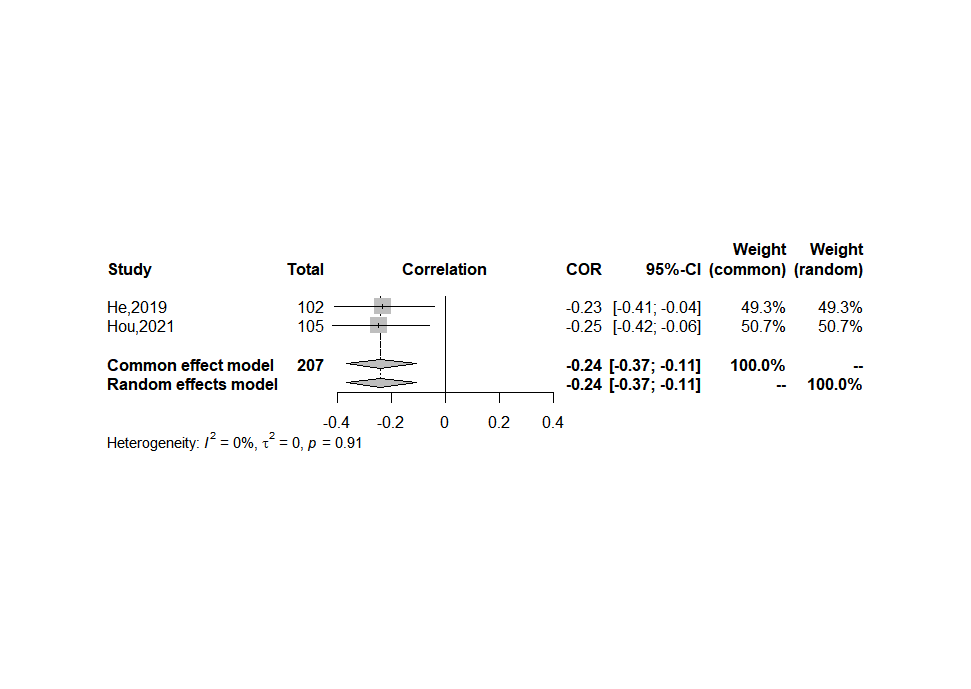


Figure 20 Forest plot: the summary z value with corresponding 95% CIs for the correlation between attachment avoidance of caregiver and caregiver preparedness.


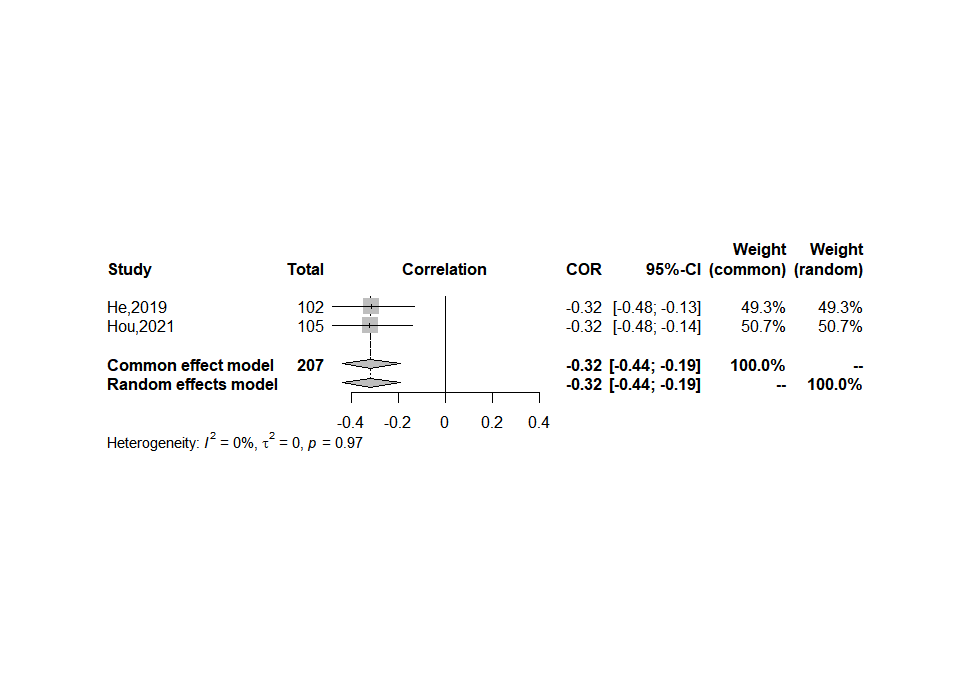
Figure 21 Forest plot: the summary z value with corresponding 95% CIs for the correlation between attachment anxiety of caregiver and caregiver preparedness.
